# Study on HIV/AIDS knowledge, sexual attitudes, sexual behaviors, and preventive services among young students in Chongqing, China

**DOI:** 10.3389/fpubh.2022.982727

**Published:** 2022-10-10

**Authors:** Liyuan Qing, Yunna Wang, Tong Yang, Xinrui Chen, Meng Zhang, Qingqing Bu, Bo Tan, Dan Deng

**Affiliations:** Department of Health Statistics, School of Public Health, Chongqing Medical University, Chongqing, China

**Keywords:** China, students, sexual attitude, sexual behavior, HIV/AIDS knowledge, education

## Abstract

**Objectives:**

This study aimed to describe the HIV/AIDS knowledge, sexual attitudes, and sexual behaviors of young students (14–25 years) in Chongqing, China, and to examine their use of preventive services and related factors.

**Method:**

A cross-sectional study was conducted with students from 67 schools from December 2019 to June 2021. The chi-square test, non-parametric test to assess the differences between respondents with different characteristics in the above aspects. Additionally, univariate and multivariate logistic regression analyses were conducted to examine factors associated with the awareness of HIV/AIDS knowledge. Confidence intervals without crossover or *P* < 0.05 was considered significant.

**Results:**

A total of 31,782 participants were included in the study. A total of 62.62% of the respondents were considered to be aware of basic HIV/AIDS knowledge, including 78.44%, 62.15%, and 52.08% in undergraduate colleges, junior colleges and secondary vocational schools, respectively. In total, 60.96% of males and 29.42% of females accepted premarital sex, 15.49% of males and 6.18% of females reported being sexually experienced, and only 57.18% of the students used condoms every time they had sex. The percentage of condom use among students who were aware of HIV/AIDS knowledge was higher than that among students who were not aware. A total of 85.60% of the students thought they needed HIV/AIDS prevention and control knowledge, and 94.60% thought more health education activities on HIV/AIDS prevention needed to be conducted in schools. Entrance education, lectures or consultation hotlines, WeChat official accounts and other media platforms were rated as the most acceptable HIV/AIDS prevention education methods.

**Conclusion:**

These respondents lacked knowledge about HIV/AIDS, and an increasing number of students show an open attitude toward premarital sex and engage in risky sexual behaviors. It is urgent to enhance and widely disseminate comprehensive HIV/AIDS and sexual health education in multiple formats.

## Introduction

Acquired immunodeficiency syndrome (AIDS) is a chronic progressive infectious disease caused by the human immunodeficiency virus (HIV). According to the latest Word Health Organization (WHO) estimates, an estimated 38.40 million people were living with HIV globally at the end of 2021 (1). Youth are a population greatly impacted by HIV both nationally and globally, the Joint United Nations Programme on HIV/AIDS (UNAIDS) indicates that two out of every seven new HIV infections globally in 2019 were among young people (15–24 years) (2). In the United States, 21% of new HIV diagnoses in 2019 were among young people (aged 13–24) and Almost half of young people with HIV do not know they have the virus (3). In China, there were 1.05 million people living with HIV, with 351,000 deaths reported (4). The number of newly reported infection among young students increasing each year, while decline globally (5). According to statistics from the Chinese Centers for Disease Control and Prevention, 23,307 young students newly reported HIV/AIDS from 2010 to 2019. The number of HIV/AIDS cases increased from 794 in 2010 to 3,422 in 2019, and 98.2% of cases occurred through sexual transmission (6).

A lack of sexual knowledge leads to not only an increased infection rate of HIV/AIDS (7) but also other reproductive health diseases, accidental pregnancy and abortion. According to data from the National Health Commission of People's Republic of China, the number of induced abortions in 2019 and 2020 reached 9.76 million and 8.96 million, respectively (8). A survey of undergraduate students aged 18–25 from 130 universities in China showed that among female students in the surveyed schools, abortion rates were as high as 32.87% (9). The National Health Commission and 10 other departments jointly formulated the Implementation Plan to Combat AIDS Transmission (2019–2022) and required young students to have an awareness rate of at least 95% of AIDS prevention and control knowledge (10).

Chongqing is one of the four municipalities directly under the central government in China. It is one of the places with a high incidence of AIDS in China (11), with the cumulative number of HIV/AIDS among young students in Chongqing ranking third from 2010 to 2019 (6). To our knowledge, there is still a shortage of surveys on HIV/AIDS knowledge, sexual attitudes and behaviors among young students in Chongqing. Therefore, this study aimed to describe sexual attitudes, sexual behavior, knowledge of HIV/AIDS and the use of prevention services among young students in Chongqing, China.

## Materials and methods

### Investigation subjects and quality control

There are 26 undergraduate colleges, 43 junior colleges (12), and 125 secondary vocational schools in Chongqing (13). This study adopted a multistage cluster sampling method. First, convenience sampling was used to select 1–6 schools in each district and county. Second, at least one class was selected randomly from each school. Finally, 26 municipal districts and 10 counties were investigated (only 2 counties had not been investigated), including 21 undergraduate colleges, 17 junior colleges, and 29 secondary vocational schools, with a total of 34,479 students. The survey was based on educational activities organized by the Chongqing Municipal Education Commission, Chongqing Municipal Health Commission, Chongqing Health Education Institute, and Primary and Secondary School Health Care Institute from December 2019 to June 2021. Before the start of the mission, all respondents completed the self-administered questionnaire by scanning the two-dimensional code with WeChat or paper questionnaire.

Methods to ensure the authenticity of the questionnaire were as follows: (1) Before the survey, the project investigators conducted unified training, fulfilled all requirements and took precautions. (2) The purpose of the survey and its anonymity and voluntary nature were fully communicated prior to the survey. (3) Questionnaires from the same school were required to be completed within a fixed time. (4) Electronic questionnaires required all questions to be completed before submission, and repeat submissions from the same IP address were not accepted. All paper questionnaires were checked on the spot and collected. (5) The questionnaires were carefully checked for completion, and those with large numbers of omissions, those with the same option selected for multiple consecutive questions, and those that were incorrectly completed were screened out.

The studies involving human participants were reviewed and approved by the Ethics Committee of Chongqing Medical University.

### Study variables and measurements

Basic demographic characteristics regarding the students' school, age, sex, nationality, registered permanent residence, major, and monthly expenses were collected.

HIV/AIDS knowledge was assessed with a 8-item questionnaire consistent with the National AIDS Sentinel Surveillance Questionnaire (2017 edition) (14). Each item was measured *via* a yes/no/uncertain format, and only the correct response scored 1 point. Each respondent was considered to be “aware” if he or she scored 6 points (14).

Sexual attitude referred to whether or not to accept sexual behavior, with options from 1 (completely unacceptable) to 5 (completely acceptable). The score lower than 3 were recoded as “unacceptable,” and score of 3 and higher were recoded as “acceptable.”

Sexual behavior included whether or not have had sex, the age of sexual debut, who you have sex with (spouse, heterosexual romantic partner, commercial sex with monetary transactions, casual sex without monetary transactions, same-sex, others), how often you use condoms (every time, often, occasionally, never) and why you don't want to use them.

Prevention service usage included sources of HIV/AIDS and sexual knowledge, acceptable access to obtain knowledge about AIDS prevention, whether actively inquired about AIDS, Whether they thought they need to gain some knowledge about AIDS and whether they thought it was necessary to increase HIV prevention health education in schools [1 (completely necessary) to 5 (totally unnecessary), the score of 3 and lower recoded as “necessary,” and higher than 3 were recoded as “unnecessary”], whether they had received AIDS prevention services at school in the last year, whether tested for HIV.

### Statistical methods

Mplus 8.0 was used for confirmatory factor analysis (CFA) to test the structural validity of HIV/AIDS knowledge scale, and the parameter estimation method was robust minimum weighted double multiplication (WLSMV). Kuder-Richardson Formula 20 (KR-20), a special case of Cronbach α for dichotomous response options, was used for the knowledge scale; values ≥0.7 were considered acceptable (15, 16). The internal consistency reliability calculated by the Kuder Richardson formula 20 (KR-20). Frequencies, percentages and their 95% confidence intervals were used to describe the demographic variables, HIV/AIDS knowledge awareness, sexual attitudes and behaviors, and Prevention services usage. SPSS 25.0 was used for the chi-square test, non-parametric test to assess the differences between respondents with different characteristics in the above aspects. Additionally, univariate and multivariate logistic regression analyses were conducted to examine factors associated with the awareness of HIV/AIDS knowledge. All tests were two-sided, and confidence intervals without crossover or *P* < 0.05 was considered significant.

## Results

### Subjects and demographic characteristics

Finally, a total of 34,479 participants completed the questionnaire, including 32,462 electronic questionnaires from 58 schools and 2017 paper questionnaires from 9 schools. Some questionnaires were excluded because of too short or too long to fill in, multiple questions to choose the same option consecutively or unanswered, age <14 or more than 25, the school was not on the list of the survey, etc. Finally, there were 31,782 valid questionnaires. Two thousand five hundred twenty electronic questionnaires were excluded, for an effective rate of 92.24%. One hundred seventy-seven paper questionnaires were excluded, for an effective rate was 91.22%. The demographic characteristics of the final subjects are shown in Table 1.

**Table 1:** Demographic data of young students (*n* = 31,782).

**Item**	**Category**	**Number**	**Percentage**
Sex	Male	14,391	45.28
	Female	17,391	54.72
Nationality	Minority	4,774	15.02
	Han	27,008	84.98
Registered permanent residence	Rural	22,786	71.69
	Urban	8,996	28.31
Age (years)	14~	660	2.08
	16~	8,611	27.09
	18~	9,954	31.32
	20 ~ 25	12,557	39.51
Major	Non-medical	29,701	93.45
	Medical	2,081	6.55
Monthly expenses	Under 1,000 yuan	13,108	41.24
	1,000~	16,540	52.04
	2,000~	1,519	4.78
	3,000 yuan and above	615	1.94
School type	Secondary vocational	8,623	27.13
	Junior colleges	16,906	53.19
	Undergraduate colleges	6,253	19.68

### Reliability and validity test

**Figure 1:**
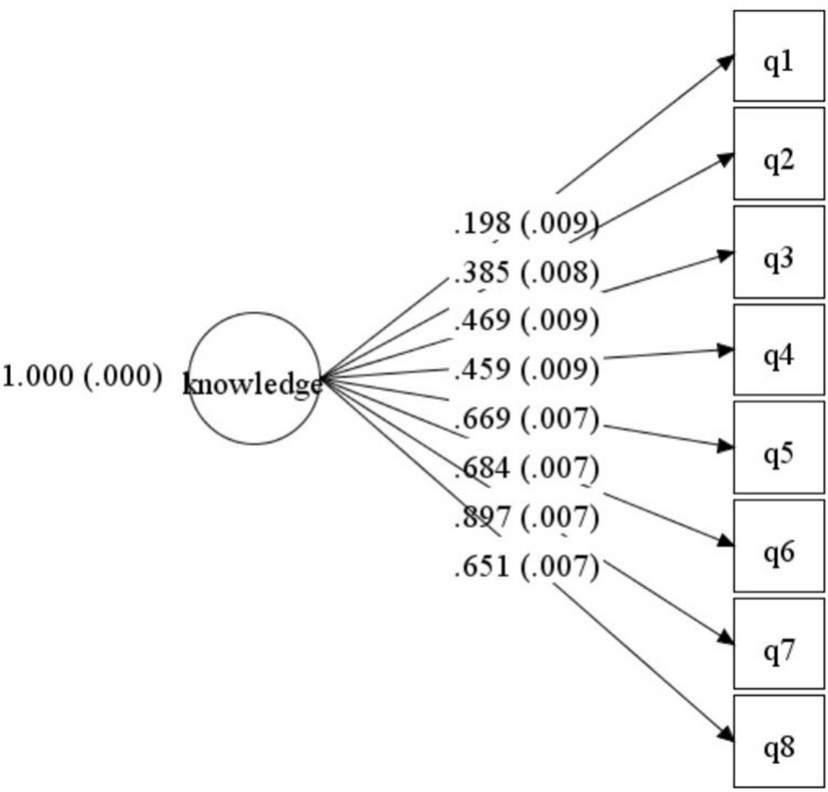
Structural validity of the HIV/AIDS knowledge questionnaire.

The reliability and validity of the measure of basic HIV/AIDS knowledge were tested. KR-20 was 0.764. The results of CFA showed that χ^2^ = 1,523.154, *df* = 20, χ^2^/*df* = 76.158, comparative fit index (CFI) = 0.956, Tucker-Lewis index (TLI) = 0.938, Standardized Root Mean Square Residual (SRMR) = 0.046, the root mean square error of approximation (RMSEA) = 0.049. Except for the first item (0.198), all factor loadings were >0.35 (Figure 1). Since all HIV/AIDS knowledge questions were selected from the National AIDS Sentinel Surveillance Questionnaire, all questions were retained for comparison with other studies. The results showed that this part had acceptable reliability and validity.

### HIV/AIDS knowledge awareness

A total of 19,903 (62.62%; 95% CI: 62.09–63.16%) students were considered aware. there were 5,191 (16.33%; 95% CI: 15.93–16.74%) students answered all the questions correctly. Among them, the awareness percentage of the importance of actively seeking AIDS detection and counseling after high-risk behaviors was the highest (87.60%), while the awareness percentage for each other knowledge item was lower than 80% (Table 2).

**Table 2:** Young students' awareness of HIV/AIDS knowledge (*n* = 31,782).

**Question**	**Correct**	**Incorrect**	**Have no idea**	**Awareness percentage [%, (95% CI)]**
q1: AIDS is an incurable serious infectious disease	21,747	6,764	3,271	68.43 (67.91–68.94)
q2: At present, the prevalence of AIDS among young students in China is growing rapidly. Male homosexual sex is the main mode of transmission, followed by heterosexual sex	17,168	7,167	7,447	54.02 (53.47–54.57)
q3: It is impossible to judge whether a person is infected with HIV/AIDS by appearance	24,298	2,609	4,875	76.45 (75.99–76.92)
q4: Daily life and learning contact will not cause one to be infected with HIV/AIDS	24,513	3,508	3,761	77.13 (76.67–77.59)
q5: Sticking to the correct use of condoms can reduce the risk of infection and transmission of HIV/AIDS	23,635	2,625	5,522	74.37 (73.89–74.85)
q6: The use of new drugs (such as methamphetamine, ecstasy, k powder) will increase the risk of HIV/AIDS infection	23,350	2,139	6,293	73.47 (72.98–73.95)
q7: After high-risk behaviors (needle sharing drug abuse, unsafe sexual behavior, etc.), take the initiative to seek AIDS testing and counseling	27,842	623	3,317	87.60 (87.24–87.97)
q8: The rights and interests of people living with AIDS such as enrolment, employment, marriage are protected by Chinese laws	20,072	1,552	10,158	63.16 (62.62–63.69)

Univariate analysis showed that the awareness percentage of female students, students of Han nationality, registered city residents and medical students was higher. The awareness percentage increased with age; in addition, it increased with monthly expenditure when the expenditure was < 3,000 yuan but decreased when the monthly expenditure was 3,000 yuan or more. The awareness percentages of undergraduate colleges, junior colleges, and secondary vocational schools were 78.44%, 62.15%, and 52.08%, respectively (Table 3).

**Table 3:** HIV/AIDS knowledge among students with different characteristics (*n* = 31,782).

**Variables**		**Number**	**Awareness percentage (%)**	***χ^2^***	***P***
Sex	Male	14,391	61.24	21.517	<0.001
	Female	17,391	63.77		
Nationality	Minority	4,774	56.68	84.727	< 0.001
	Han	27,008	63.67		
Registered permanent residence	Rural	22,786	60.41	167.856	<0.001
	Urban	8,996	68.22		
Age (years)	14~	660	47.88^a^	767.118	<0.001
	16~	8,611	51.33^a^		
	18~	9,954	65.78^b^		
	20 ~ 25	12,557	68.64^c^		
Major	Nonmedical	29,701	62.31	18.921	<0.001
	Medical	2,081	67.08		
Monthly expenses	Under 1,000 yuan	13,108	56.74^a^	342.215	<0.001
	1,000~	16,540	66.61^b^		
	2,000~	1,519	70.11^c^		
	3,000 yuan and above	615	62.28^b^		
School type	Secondary vocational	8,623	52.08^a^	1,079.532	<0.001
	Junior colleges	16,906	62.15^b^		
	Undergraduate colleges	6,253	78.44^c^		

a, b, and c are the results of Bonferroni pairwise comparison, and the same letter represents no significant difference (P < 0.05). The difference represents statistical significance (P > 0.05).

The correlation coefficient between variables was calculated before the multifactor analysis. There was a strong correlation between the variables of age, monthly expenditure, and school type (Table 4). The absolute values of correlation coefficients between other variables were < 0.25.

**Table 4:** Spearman correlation analysis among the main variables.

	**Age (years)**	**Monthly expenses**	**School type**
Age (years)	1	0.291^**^	0.583^**^
Monthly expenses	0.291^**^	1	0.389^**^
School type	0.583^**^	0.389^**^	1

** P < 0.001.

According to the results of the correlation analysis, only school type was selected as an independent variable among age, monthly expenses and school type, and other factors were included in the independent variable to construct a multifactor logistic regression model. We found that nationality, household registration, medical specialty and school type were independent related factors. Students of Han nationality (OR: 1.294; 95% CI: 1.214–1.379), registered city residents (OR: 1.158; 95% CI: 1.097–1.222), and students with a medical specialty (OR: 1.296; 95% CI: 1.177–1.427) were more likely to be aware of HIV/AIDS knowledge. Compared with students of secondary vocational schools, students of junior colleges (OR: 1.441; 95% CI: 1.365–1.520) and undergraduate colleges (OR: 3.181; 95% CI: 2.950–3.430) were more likely to be aware of HIV/AIDS knowledge (Table 5).

**Table 5:** Logistic regression analysis of HIV/AIDS knowledge among young students (*n* = 31,782).

**Variables**	**Control**	**Univariate analysis**		**Multivariate analysis**	
		**OR (95% CI)**	***P***	**OR (95% CI)**	***P***
**Sex**					
Female	Male	1.114 (1.064–1.166)	<0.001		
**Nationality**					
Han	Minority	1.340 (1.259–1.426)	<0.001	1.294 (1.214–1.379)	<0.001
**Registered permanent residence**					
Urban	Rural	1.407 (1.336–1.481)	<0.001	1.158 (1.097–1.222)	<0.001
**Age (years)**					
16~	14~	1.148 (0.980–1.345)	0.008		
18~		2.093 (1.787–2.452)	<0.001		
20~25		2.383 (2.036–2.788)	<0.001		
**Major**					
Medical	Non-medical	1.233 (1.122–1.355)	<0.001	1.296 (1.177–1.427)	<0.001
**Monthly expenses**					
1,000~	Under 1,000 yuan	1.521 (1.450–1.594)	<0.001		
2,000~		1.788 (1.594–2.006)	<0.001		
3,000 yuan and above		1.258 (1.065–1.487)	0.007		
**School type**					
Junior colleges	Secondary vocational	1.511 (1.434–1.592)	<0.001	1.441 (1.365–1.520)	<0.001
Undergraduate colleges		3.348 (3.110–3.604)	<0.001	3.181 (2.950–3.430)	<0.001

### Sexual attitudes

In total, 13,889 (43.70%; 95% CI: 43.20–44.25%) students accepted premarital sex. 60.96% (95% CI: 60.16–61.76%) of male students and 29.42% (95% CI: 28.74–30.09%) of female students, respectively. The acceptance percentages of premarital sex among students who were aware and unaware of HIV/AIDS knowledge were 47.33 and 37.61%, respectively (χ^2^ = 285.782, *P* < 0.001). In addition to men in the undergraduate colleges and women in secondary vocational schools, the acceptance percentage of the aware HIV/AIDS knowledge group was higher than that of the unaware group (Table 6).

**Table 6:** Young students' attitudes toward premarital sexual behavior (*n* = 31,782).

**School type**	**Sex**	**Knowledge of HIV/AIDS**	**Number**	**Acceptance percentage**	
				**%**	**95% CI**
Secondary vocational	Male	Unaware	2,493	45.17	43.21–47.12
		Aware	2,643	50.28	48.38–52.19
		Total	5,136	47.80	46.43–49.17
	Female	Unaware	1,639	10.74	9.24–12.24
		Aware	1,848	13.04	11.50–14.58
		Total	3,487	11.96	10.88–13.04
Junior colleges	Male	Unaware	2,645	61.29	59.43–63.14
		Aware	4,359	68.71	67.33–70.09
		Total	7,004	65.91	64.79–67.02
	Female	Unaware	3,754	24.80	23.42–26.18
		Aware	6,148	33.56	32.39–34.74
		Total	9,902	30.24	29.33–31.14
Undergraduate colleges	Male	Unaware	440	70.68	66.41–74.95
		Aware	1,811	76.81	74.86–78.75
		Total	2,251	75.61	73.84–77.39
	Female	Unaware	908	33.37	30.30–36.44
		Aware	3,094	45.31	43.56–47.07
		Total	4,002	42.60	41.07–44.14

### Sexual behaviors

In total, 3,304 students (10.40%; 95% CI: 10.06–10.73%) had had sex. A total of 15.49% (95% CI: 14.90–16.08%) of male and 6.18% (95% CI: 5.82–6.54%) of, female respectively, 12.75% of students in undergraduate colleges, 12.01% of students in junior colleges, and 5.52% of students in secondary vocational institutions, respectively. The incidence percentages of sexual behavior in students who were aware and unaware of HIV/AIDS were 11.46 and 8.61%, respectively (χ^2^ = 64.809, *P* < 0.001, Table 7).

**Table 7:** Sexual behavior of young students (*n* = 31,782).

**School type**	**Sex**	**Knowledge of HIV/AIDS**	**Number**	**Had sex**	
				**%**	**95% CI**
Secondary vocational	Male	Unaware	2,493	8.42	7.33–9.51
		Aware	2,643	7.98	6.95–9.02
		Total	5,136	8.20	7.45–8.95
	Female	Unaware	1,639	1.40	0.83–1.97
		Aware	1,848	1.73	1.14–2.33
		Total	3,487	1.58	1.16–1.99
Junior colleges	Male	Unaware	2,645	16.22	14.81–17.63
		Aware	4,359	20.90	19.69–22.11
		Total	7,004	19.13	18.21–20.05
	Female	Unaware	3,754	5.30	4.58–6.02
		Aware	6,148	8.00	7.32–8.68
		Total	9,902	6.98	6.48–7.48
Undergraduate colleges	Male	Unaware	440	22.95	19.01–26.90
		Aware	1,811	20.27	18.41–22.12
		Total	2,251	20.79	19.11–22.47
	Female	Unaware	908	6.72	5.09–8.35
		Aware	3,094	8.66	7.67–9.65
		Total	4,002	8.22	7.37–9.07

Among the 3,111 people who provided a response regarding the age of sexual debut, 182 (5.85%) reported an age of 14 and below, 904 (29.06%) an age of 15–17 years old, and 2,025 (65.09%) an age of 18 and older; the median age of sexual debut was 18.

Among the 3,311 students who responded regarding his or her sexual partner, 3,307 (99.88%) had sex with their spouse or heterosexual romantic partner, 139 (4.20%) had commercial sex with monetary transactions, 236 (7.13%) had casual sex without monetary transactions, and 139 (4.20%) had same-sex sexual behaviors. A total of 101 of 2,209 males (4.57%; 95% CI: 3.70–5.44%) had same-sex sexual activity.

Among the 3,302 students who responded regarding the frequency of condom use, only 1,888 (57.18%; 95%: 55.49–58.87%) used condoms every time, and 301 (9.12%; 95% CI: 8.13–10.10%) never used condoms. The results showed that students who had had commercial, temporary, same-sex, or other sex were more inclined to not use condoms (Table 8). A total of 60.89% of students who were aware of HIV/AIDS knowledge used condoms every time, which was 11.96 percentage points higher than the unaware group (Table 9).

**Table 8:** Frequency of condom use during sex.

**Sexual partner**	**Every time *n* (%)**	**Often *n* (%)**	**Occasionally *n* (%)**	**Never *n* (%)**	**Average rank**	**Rank sum**	***z***	***P***
Spouse, heterosexual romantic partner (*n* = 2,826)	1,688 (59.73)	516 (18.26)	445 (15.75)	177 (6.26)	1,592.06	4,499,167.0	9.744	<0.001
Commercial, casual, same-sex, Other (*n* = 476)	200 (42.02)	76 (15.97)	76 (15.97)	124 (26.05)	2,004.38	954,086.0		
Total (*n* = 3,302)	1,888 (57.18)	592 (17.93)	521 (15.78)	301 (9.12)				

**Table 9:** Frequency of condom use during sexual behavior among those aware and unaware of HIV/AIDS knowledge.

**Knowledge of HIV/AIDS**	**Every time *n* (%)**	**Often *n* (%)**	**Occasionally *n* (%)**	**Never *n* (%)**	**Average rank**	**Rank sum**	***z***	***P***
Unaware (*n* = 1,024)	501 (48.93)	184 (17.97)	192 (18.75)	147 (14.35)	1,821.05	1,864,758.5	−7.648	<0.001
Aware (*n* = 2,278)	1,387 (60.89)	408 (17.91)	329 (14.44)	154 (6.76)	1,575.28	3,588,494.5		
Total (*n* = 3,302)	1,888 (57.18)	592 (17.93)	521 (15.78)	301 (9.12)				

In 27.19% of cases, the reason for not using condoms was that the respondents or their sexual partners were reluctant; in 23% of girls, the reason was their sexual partners were reluctant to use condoms. For 24.75% of respondents, the reason for not using condoms was because they were not available for purchase, they were too expensive, they did not know where to buy them, or they felt embarrassed to buy them. A total of 13.93% of respondents used other contraceptive measures (Table 10).

**Table 10:** Reasons for reluctance to use condoms (*n* = 1,414).

**Reasons**	**Number of cases**	**Response percentage (%)**	**Percent of cases (%)**
The other partner was unwilling	278	11.10	19.66
The respondent was unwilling	403	16.09	28.50
Not available for purchase	223	8.90	15.77
Too expensive	135	5.39	9.55
Don't know where to buy	61	2.44	4.31
Feel embarrassed to buy	201	8.02	14.21
Used other contraceptive measures	349	13.93	24.68
No need to use	259	10.34	18.32
Forgot	267	10.66	18.88
Never used	107	4.27	7.57
Other	222	8.86	15.70
Total	2 505	100	177.16

The number of cases is the number of people who choose this option, the response percentage is (the number of people who chose this option/the number of times all options were selected) *100%, and the percentage of cases is (the number of people who chose this option/the number of people who answered the question) *100%.

### Prevention services usage

The sexual knowledge of young students in the investigated schools mainly came from classroom education, the internet, chatting with friends, TV media, books or magazines, and family education (Table 11).

**Table 11:** Main sources of sexual knowledge (*n* = 31,782).

**Sources**	**Number of cases**	**Response percentage (%)**	**Percent of cases (%)**
Classroom education	22,212	17.58	69.89
Internet	21,475	17.00	67.57
Chatting with friends	15,729	12.45	49.49
TV media	15,594	12.34	49.07
Books or magazines	15,161	12.00	47.70
Family education	14,378	11.38	45.24
Romance novel	6,978	5.52	21.96
Love action film	6,096	4.83	19.18
Self-exploration, social practice	5,954	4.71	18.73
Other	2,762	2.19	8.69
Total	126,339	100	397.52

HIV/AIDS knowledge mainly came from the internet and related thematic education organized by schools, television, or radio (Table 12).

**Table 12:** Main sources of HIV/AIDS knowledge (*n* = 31,774).

**Sources**	**Number of cases**	**Response percentage (%)**	**Percent of cases (%)**
Internet	26,814	21.95	84.39
Related thematic education organized by schools	25,829	21.15	81.29
Television or radio	24,197	19.81	76.15
Teachers or classmates	16,947	13.87	53.34
Books or newspapers	16,577	13.57	52.17
Families	8,328	6.82	26.21
Other	3,459	2.83	10.89
Total	122,151	100	384.44

HIV/AIDS prevention education when entering school, special lectures or telephone consultations, WeChat official accounts and other media platforms, and HIV/AIDS prevention publicity activities carried out by student associations were reported to be the most acceptable methods for HIV/AIDS prevention publicity and education (Table 13).

**Table 13:** Main sources of HIV/AIDS knowledge that students were willing to accept (*n* = 31,773).

**Sources**	**Number of cases**	**Response percentage (%)**	**Percent of cases (%)**
Education when entering school	24,156	21.43	76.03
Special lectures or telephone consultations	22,800	20.23	71.76
WeChat official account and other media platforms	20,568	18.25	64.73
Publicity activities carried out by student associations	16,756	14.86	52.74
Teaching of elective courses in the whole school	15,551	13.80	48.94
Peer education	10,692	9.48	33.65
Other	2,204	1.95	6.94
Total	112,727	100	354.79

A total of 15,200 (47.83%; 95% CI: 47.28–48.38%) students had actively inquired about AIDS, 48.47% (95% CI: 47.64–49.30%) of whom had accepting attitudes toward premarital sex, 47.33% (95% CI: 46.59–48.06%) of whom did not have accepting attitudes, 53.69% (95% CI: 51.99–55.39%) of whom had had sex, and 47.15% (95% CI: 46.57–47.73%) of who had not had sex. Most of the students thought they needed knowledge about HIV/AIDS prevention and treatment and it was necessary to conducted more HIV prevention health education in schools. A total of 20,651 (64.98%; 95% CI: 64.45–65.50%) had received HIV/AIDS prevention publicity services in school in the last year. Only 3,399 (10.69%; 95% CI: 10.35–11.03%) had received HIV testing services. Those students who were aware of basic HIV/AIDS knowledge received more preventive services (Table 14).

**Table 14:** Use of HIV/AIDS prevention services in the past (*n* = 31,782).

**Service**	**Knowledge of HIV/AIDS**	**Number**	**%**	**95% CI**
**Thought some knowledge of HIV/AIDS prevention and control was needed**				
	Unaware	9,964	83.88	83.22–84.54
	Aware	17,242	86.63	86.16–87.10
	Total	27,206	85.60	85.22–85.99
**Thought it was needed to increase HIV/AIDS prevention health education activities in schools**				
	Unaware	10,971	92.36	91.88–92.83
	Aware	19,094	95.94	95.66–96.21
	Total	30,065	94.60%	94.35–94.85
**Actively inquired about HIV/AIDS knowledge**				
	Unaware	4,516	38.02	37.14–38.89
	Aware	10,684	53.68	52.99–54.37
	Total	15,200	47.83	47.28–48.38
**Received HIV/AIDS prevention publicity service in school in the last year**				
	Unaware	6,310	53.12	52.22–54.02
	Aware	14,341	72.05	71.43–72.68
	Total	20,651	64.98	64.45–65.50
**Had HIV testing before**				
	Unaware	1,230	10.35	9.81–10.90
	Aware	2,169	10.90	10.46–11.33
	Total	3,399	10.69	10.35–11.03

## Discussion

The results showed that the awareness percentage of basic HIV/AIDS knowledge among young students was 62.62%. Compared to other provinces, the present findings were lower than the findings of Cong Zhao in 15 universities in Wuhan (90.8% for men who have sex with men (MSM) and 64.6% for non-MSM) (17) and the 2019 survey results for Beijing, Shenzhen and Kunming (85.6%) (18) but higher than the 2016 survey results for freshmen at Qinghai University (48.59%) (19). The differences are partly due to some of the differences in the issues addressed in this study and other studies as well as to the inclusion of a higher proportion of younger age groups in this study than in others; in addition, the differences in awareness percentages between provinces and municipalities may be related to the approach and intensity of HIV/AIDS prevention education efforts in different regions and schools. However, it is certain that the awareness percentage among students of different genders, professions, ages and schools did not reach 95%, which is the requirement of the Implementation Plan to Curb the Spread of HIV/AIDS (2019–2022) (8). There is not enough awareness, especially about the dangers of HIV (q1 awareness percentage: 68.43%, even 21.28% of the respondents thought AIDS can be cured; q2 knowledge percentage: 54.02%), indicating that students in the surveyed schools are not fully aware of the seriousness of HIV/AIDS, the main modes of transmission among young people and the risks of unprotected sex behavior.

Students of Han nationality were more likely to be aware of HIV/AIDS, and this may be partly due to cultural factors. Ethnic minority students may have language barriers in receiving HIV education and be more likely to grow up in rural areas where they have fewer chances to obtain HIV/AIDS knowledge (20). Medical majors had higher awareness percentages, which was consistent with other studies. This may be attributed to the fact that medical students receive extensive medical courses, including AIDS-related education. Compared to students in undergraduate colleges (78.44%) and junior colleges (62.15%), students in secondary vocational institutions (52.08%) had less knowledge of HIV/AIDS, which might be related to the different age distributions of students, and higher education might be associated with increased probability of mass media and internet exposure leading to higher awareness of HIV (21). However, positive changes in the knowledge and behaviors of secondary vocational school students are still possible (22). Targeted HIV/AIDS education and interventions should be strengthened, and all youth should know how HIV is transmitted and prevented, understand what puts them at risk for HIV, and be tested if they are at risk.

It is evident that males are generally more likely to be open to premarital sex. Those students who were aware of HIV/AIDS knowledge were more likely to be open to premarital sex and to have had sexual intercourse. Perhaps these students were more proactive in learning about sexual health. This was confirmed by the question about whether they actively sought knowledge of HIV/AIDS. This study revealed that 10.40% of participants had had sexual experiences, including 12.75% in undergraduate colleges and 12.01% in junior colleges; these percentages were higher than the results of other papers published in 2021 (11.98%) (23) and 2013 (9%) (24). Moreover, the median age at first sexual intercourse was 18, earlier than the results from 49 universities in 2016 (mean age was 20.14) (25); 34.91% of students and their sexual debut before the age of 18, higher than the results of a national survey of 130 schools in 2015 (23.8%) (26). Unlike the United States, which decrease in the prevalence of ever having had sexual intercourse in the past (27), students exhibit open attitudes toward sex and that the age of sexual debut is declining in China (28). This phenomenon is not confined to China (29). Early initiation of sexual intercourse is associated with multiple partners, infrequent condom use, unwanted pregnancies, unsafe abortions, sexually transmitted infections and HIV infection (30, 31). There was also a high prevalence of unsafe sex among those who had sexual intercourse (only 57.18% used condoms every time they had sex, 4.20% had commercial sex with monetary transactions, 7.13% had casual sex without monetary transactions, and 4.57% of men had homosexual sex). A total of 60.89% of the students who were aware of HIV/AIDS knowledge used condoms every time they had sex, which was higher than that in those who were unaware (48.93%). It can be seen that mastering HIV/AIDS knowledge helps to engage in safe sex behaviors.

Students in this study acquired sexual knowledge mainly from school classroom education (69.89%), while a 2015 survey stated that 44.4% of students reported that they had never received sexuality education from school (26). Therefore, schools have assumed increasingly important responsibilities in sex education. However, 24.75% of the students did not use condoms because they thought they were not available or were too expensive, they did not know where to buy them or they felt embarrassed to buy them. Twenty-three percent of the girls did not use condoms because their sex partner were unwilling to use them. Although sex education was formally introduced into the school curriculum in 1988, it is still not comprehensive enough and lacks training in sex education for teachers (32). Limited educational resources cannot meet the practical needs of young people, such as instruction in the prevention of sexually transmitted infections and pregnancy, how to use condoms, and consent issues (33). As evidenced by a sharp increase in the sexual activity of younger generations (32), traditional abstinence education no longer meets contemporary needs (34–36). In addition, 64.98% of students had received HIV/AIDS prevention and publicity services in school in the past year, 85.60% thought they needed HIV/AIDS prevention and treatment knowledge, and 94.60% thought it was necessary to increase HIV/AIDS prevention and health education activities; these findings suggest that there is still a lack of HIV/AIDS knowledge and publicity education in schools. Comprehensive HIV/AIDS and sexual health education needs to be developed and widely disseminated in China and should cover condom use, how to prevent sexually transmitted diseases and pregnancy, sexual consent, etc.

Although a completely random sample was not employed in this study, a total of 67 institutions in Chongqing were involved in this study, accounting for 34.54% of the total number of schools, and the sample size was large enough. Therefore, the research results are still representative and can provide a reference for HIV/AIDS education and sexual health among young students nationwide.

## Data availability statement

The datasets presented in this article are not readily available because the data contains sensitive information of districts, counties, and schools. Requests to access the datasets should be directed to DD, 100079@cqmu.edu.cn.

## Ethics statement

The studies involving human participants were reviewed and approved by the Ethics Committee of Chongqing Medical University. Prior to participation, we explained the study purpose to each of the participants and emphasized that participation was voluntary and anonymous. Written informed consent to participate in this study was provided by the participants or participants legal guardian or next of kin.

## Author contributions

LQ collected, analyzed the data, and wrote the first manuscript. YW and TY designed the study and collected data. DD designed the study and revised the paper. XC, MZ, QB, and BT collected the data. All authors approved the final manuscript.

## Funding

This study was funded by Chongqing Municipal Health Commission (Grant No. X7769) and the Science and technology communication and popularization project of Chongqing Science and Technology Bureau (Grant No. cstc2020kpzx-kphdA0046).

## Conflict of interest

The authors declare that the research was conducted in the absence of any commercial or financial relationships that could be construed as a potential conflict of interest.

## Publisher's note

All claims expressed in this article are solely those of the authors and do not necessarily represent those of their affiliated organizations, or those of the publisher, the editors and the reviewers. Any product that may be evaluated in this article, or claim that may be made by its manufacturer, is not guaranteed or endorsed by the publisher.
